# Which is the best schedule of autologous blood storage for preoperative adolescent idiopathic scoliosis patients?

**DOI:** 10.1186/1748-7161-10-S2-S11

**Published:** 2015-02-11

**Authors:** Koji Tamai, Hidetomi Terai, Hiromitsu Toyoda, Akinobu Suzuki, Hiroyuki Yasuda, Shou Dozono, Hiroaki Nakamura

**Affiliations:** 1 Department of Orthopedic Surgery, Osaka City University Graduate School of Medicine, Osaka, Japan

**Keywords:** adolescent idiopathic scoliosis, autologous blood storage, autologous blood transfusion, allogeneic blood transfusion, adverse event, anemia

## Abstract

**Background:**

It is critically important for AIS patients to avoid perioperative allogeneic blood transfusions. Toward this aim, many institutes use autologous blood storage to perform perioperative transfusions. However, there is no standard timeline for collecting blood for storage. Therefore, the objective of this prospective cohort study was to compare the outcome of two different schedules for collecting autologous blood before operation in adolescent idiopathic scoliosis (AIS) patients.

**Methods:**

Inclusion criteria are AIS patients, younger than 20 years old, female, operated between 2009 and 2013 with posterior spinal fusion and instrumentation who had 1600 mL autologous blood collected before operation. A total of 61 patients were participated in this study. They were randomly divided into 2 groups based on the storage interval. Weekly group (1W-G) consisted of 30 patients with a total of 1600mL blood collected weekly beginning 4 weeks before the operation. Biweekly group (2W-G) consisted of 31 patients with a total of 1600 mL blood collected biweekly beginning 8 weeks before the operation. The instrumented levels, total bleeding, complications during blood transfusion, and hematological examinations (RBC, Hb, Hct, MCH, MCV, MCHC) were evaluated. A hematological examination was performed before blood collection, before the operation, and on postoperative days 1, 3, and 7. Vasovagal reflex (VVR) was evaluated as complications during blood drawing.

**Result:**

Mean age, height, and weight did not differ significantly between the 2 groups. There were no significant differences in instrumented levels, bleeding during operation, after operation, and collected blood during operation. With the autologous blood, allogeneic blood transfusion was completely avoided. VVR was more frequent in the biweekly group significantly (1W-G 4.2% vs 2W-G 15.3%). In terms of hematological examination, all values showed no significant differences between two groups in the pre-drawing and the pre-operation stage. However, the postoperative Hb and Hct values were higher in the weekly group. Also, MCV and MCHC showed the same behavior with higher values in the weekly group.

**Conclusion:**

A weekly schedule of autologous blood storage is better than a biweekly storage schedule.

## Introduction

Spinal fusion with instrumentation is among the most common surgical procedures that are often associated with substantial blood loss and the necessity of blood transfusion. The Nationwide Inpatient Sample (NIS) database analysis by Yoshihara [1] demonstrated that 37.9% patients who underwent spinal fusion of more than 9 vertebrae had received some kinds of transfusion during and after operation. We believe that it is very important for adolescent idiopathic scoliosis (AIS) patients to avoid perioperative allogeneic blood transfusions to avoid the risk of transmission of chronic infections. Toward this aim, our institute have regularly uses stored autologous blood for perioperative transfusions. Generally, blood collection starts 4 weeks before the operation to match with the time limit of maintaining the quality of citrate phosphate dextrose-adenine 1 (CPDA-1). According to autologous blood transfusion guidelines, the maximum permissible total amount of preoperative autologous blood is 1600 mL. [2-4], There is however, no standardised timeline for collecting blood for storage. We hypothesized that a longer period for collecting autologous blood would allow patients to have sufficient time to recover from the induced anemia preoperatively. To verify our hypothesis, we compared prospectively two different schedules for collecting autologous blood; one was to collect 1600 mL on weekly schedule, and the other was to collect on a biweekly schedule.

## Patients and methods

### Patients

**Figure 1:**
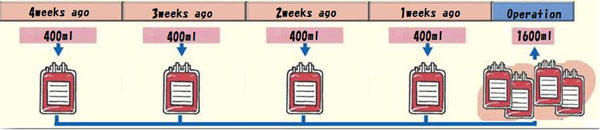
The storage schedule of 1W-G

**Figure 2:**
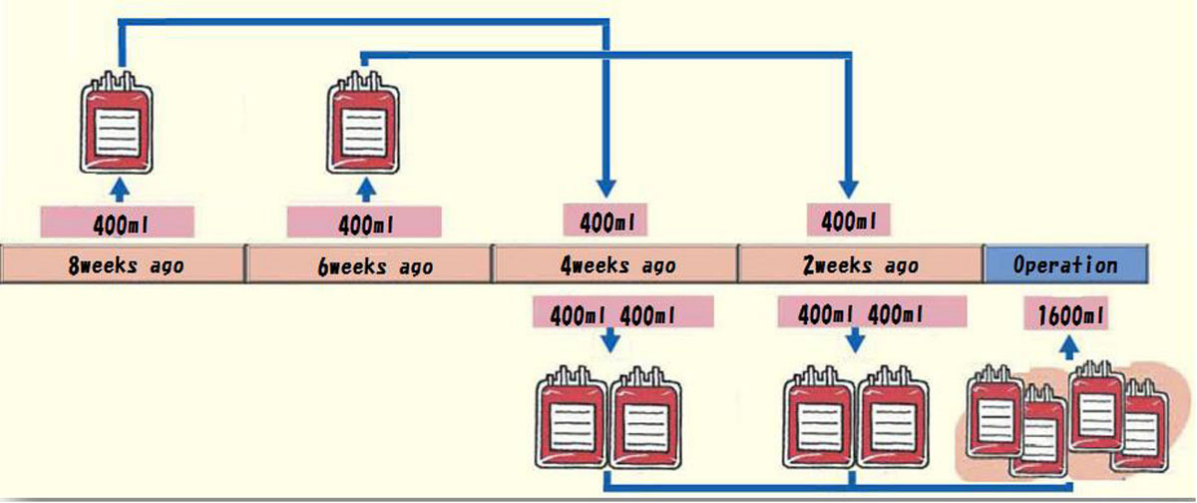
The storage schedule of 2W-G: In the latter two times, we collected 800ml per a time using 400ml of autologous blood transfusion which is collected in first and second times

Inclusion criteria of this study were AIS girls younger than 20 years old and operated between 2009 and 2013 with posterior spinal fusion and instrumentation. Male and reoperation cases were excluded. A total of 61 patients were included in this study. They were randomly divided into 2 groups based on the blood storage interval. Weekly group (1W-G) consisted of 30 patients who had 1600 mL autologous blood collected at weekly interval beginning 4 weeks before the operation (Figure 1). Biweekly group (2W-G) consisted of 31 patients who had 1600 mL autologous blood collected biweekly beginning 8 weeks before the operation. (Figure 2). 40mg of Saccharated ferric oxide and 12000IU of epoetin alfa were given to each of the collections in both groups. Mean age, height, and weight did not differ significantly between the 2 groups (Table 1). *Patients participated voluntarily in this study*, *having signed to attest their informed consent.* This research received approval from the Ethical Commission of our institute, Osaka City University Graduate School of Medicine.

**Table 1:** Preoperative patients data

	Weekly group (1W-G)	Biweekly group (2w-G)	
n	30	31	
age (year old)	15.5±3.1	15.4±2.3	N.S.(p=0.20)
height (cm)	155.8±5.9	156.1±2.3	N.S.(p=0.82)
weight (kg)	46.7±5.8	46.1±6.1	N.S.(p=0.81)

Mean±SD N.S.: not significant

### Outcome

Instrumented levels, total bleeding, complications during drawing, and hematological examinations (RBC, Hb, Hct, MCH, MCV, MCHC) were evaluated. A hematological examination was performed before drawing, before the operation, and on postoperative days 1, 3, and 7. Vasovagal reflex (VVR) was evaluated as a complication during drawing according to following grading; Grade1: Blood pressure drop or bradycardia>40/min. Grade2: blood pressure drop <90mmHg, bradycardia <40/min or unconsciousness. Grade3: convulsions or incontinence [5]. (Table 2)

**Table 2:** Grading of VVR

	Obligatory symptoms	Other symptoms
**Grade1**	blood pressure drop	pallor, cold sweats and nausea
		
	bradycardia >40/min	

**Grade2**	blood pressure drop <90mmHg	vomiting
		
	bradycardia <40/min	
		
	unconsciousness	

**Grade3**	convulsions	
		
	incontinence	

### Statistical analysis

Statistical analyses were performed with SPSS, version 12.0.1, for a personal computer (SPSS Inc., Chicago, IL). Mann–Whitney U test and Fisher’s exact test was used to analyze significant differences between these groups at each time point. Significance was set at a *P* value of 0.05.

## Results

**Figure 3:**
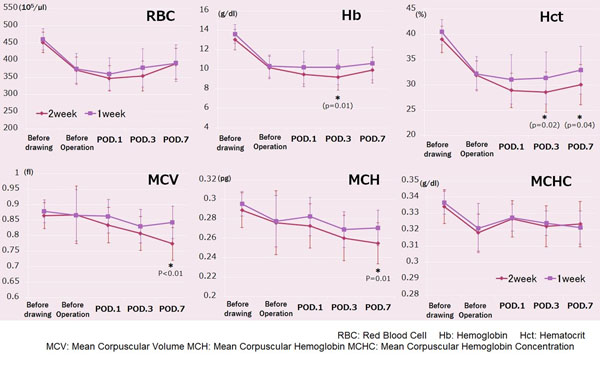
Result of hematological examination

There were no significant differences between the two groups in instrumented levels, bleeding during operation, after operation, and perioperative autologous blood collections (Table 3). All autologous blood was used peri-operatively, thus avoiding totally the need for any allogeneic blood transfusion. VVR was more frequently found in the biweekly group (1W-G 4.2% vs 2W-G 15.3%, p<0.001). (Table 4) No significant differences was found in the routine haematological tests in the pre-drawing and the pre-operation stage. However, the postoperative Hb and Hct values were higher in the weekly group (Figure 3). Also MCV and MCHC showed the same behavior with higher values in the weekly group (Figure 3).

**Table 3:** Perioperative parameter which affect to post-operative anemia

	Instrumentation	Bleeding During operation	Bleeding after operation	Collected blood During operation
1W-G	9.7±2.0 level	1316±927 ml	797±301 ml	527±403 ml
2W-G	10.5±1.7 level	1405±878 ml	643±281 ml	644±377 ml
	N.S.(p=0.14)	N.S.(p=0.70)	N.S.(p=0.07)	N.S.(p=0.10)

Mean±SD N.S.: not significant

**Table 4:** Incidence of VVR

		Grade I	Grade II	Grade III	Total
1W-G	n=30	3.3%	0.8%	0%	4.2%
	(number of times)	(4/120)	(1/120)	(0/120)	(5/120)

2W-G	n=31	10.5%	0.048	0%	15.3%
	(number of times)	(13/124)	(6/124)	(0/124)	(19/124)

		** p<0.001	** p<0.001		** p<0.001

## Discussion

We compared two schedules to collect autologous blood with the hypothesis that a longer schedule would help patients to recover faster from severe anemia after their operation, because they have enough time to recover from anemia before operation and to take nutrient like iron which is necessary for production new red blood cell. However, contrary to our hypothesis, the shorter schedule group showed faster recovery from anemia than longer group in this result. Two potential explanations for this phenomenon are the freshness of autologous blood and the behavior of the endogenic erythropoietin. Concerning the freshness of autologous blood, d’Almeida reported the viability of stored RBCs of rat and human with CPDA-1 medium [6]. They mentioned that post-transfusion viability in rat cells was 79%, 26% and 5% after 1, 2 and 4 weeks of storage respectively and human RBC deformability decreased significantly by 34% after 4 weeks of storage. In our trial, 800 mL of the blood from the biweekly group was older than that of the weekly group. This could lead to an increased amount of lower-viability blood cells in the biweekly group, resulting in lesser effectivc transfusion. Furthermore, improvement of anemia in the weekly group was found to be more rapid than biweekly group. This may be due to better stimulation of endogenic erythropoietin production in the weekly group than in the biweekly group, resulting in quick recovery of the weekly group.

In addition, biweekly group shows higher incidence of VVR than weekly group. Generally, grade 2 or 3 VVR incidence is reported in less than 1% of patients [5]. Comparing these reports, weekly group whose incidence of VVR (grade2 or 3) was 0.83% Vs the biweekly group of 4.84%.

There are some limitations in the present prospective study. First, we did not evaluate endogenic erythropoietin and iron concentrations. Secondly, the postoperative follow-up period was 7 days. Finally, although this is the first study of comparing schedule to collect autologous blood prospectively, we compared just only two schedules. Further studies should attempt to investigate further the best schedule to collect autologous blood for AIS patients.

## Conclusion

We concluded that a weekly schedule of pre-operative autologous blood storage is better than a biweekly storage schedule in terms of postoperative recovery of anemia and complications.

This is the extended abstract of IRSSD 2014 program book [7].

## Consent

Written informed consent was obtained from the patient and their parents for publication of this paper and any accompanying images. A copy of the written consent is available for review by the Editor-in-Chief of this journal.

## Competing interests

The author declares that they have no competing interests.

## Authors’ contributions

KT the corresponding author measured analysed the data and wrote the manuscript. HT collected and maintained. HT, AS and HY assisted in data analysing and interpretation. HN reviewed and edited the manuscript. All authors read and approved the final manuscript.

## Declarations

Publication charges for this collection were funded by IRSSD 2014 Sapporo.

This article has been published as part of *Scoliosis* Volume 10 Supplement 2, 2015: Research into Spinal Deformities 9: Short Papers from the IRSSD 2014 Meeting. The full contents of the supplement are available online at http://www.scoliosisjournal.com/supplements/10/S2.
